# How to engage type-2 diabetic patients in their own health management: implications for clinical practice

**DOI:** 10.1186/1471-2458-14-648

**Published:** 2014-06-25

**Authors:** Guendalina Graffigna, Serena Barello, Chiara Libreri, Claudio A Bosio

**Affiliations:** 1 Faculty of Psychology, Università Cattolica del Sacro Cuore, Largo Gemelli 1, Milan 20123, Italy

**Keywords:** Patient engagement, Disengagement, Chronic disease, Type 2 diabetes, Patient preference, Qualitative research, Diaries

## Abstract

**Background:**

Patient engagement (PE) is increasingly regarded as a key factor in the improvement of health behaviors and outcomes in the management of chronic disease, such as type 2 diabetes. This article explores (1) the reasons for disengagement of diabetic patients and their unique subjective attitudes from their experience and (2) the elements that may hinder PE in health management.

**Methods:**

29 Type-2 uncontrolled diabetes patients were asked to keep a one-week diary related to their experience of disease management, according to the narrative inquiry qualitative approach. They were interviewed to ascertain reasons for PE. The elicited narratives were subjected to interpretive content analysis.

**Results:**

The findings suggest that patients give meaning to their diabetes and its management through a complex frame of subjective experiential dimensions (cognitive/thinking, behavioral/conative and emotional/feeling), which have an impact on the spheres of daily life that are considered to be crucial in the management of diabetes (diet, physical activity, therapy, doctor-patient relationship) for each patient. These results suggest that PE develops along a continuum featuring four subsequent phases (blackout, arousal, adhesion, eudaimonic project). Several unmet needs related to the different phases of the PE continuum were discovered and illuminated possible types of support.

**Conclusions:**

Our findings appear to confirm some features of PE detected by previous research, such as a behavioral component. We were also able to shed light on the synergic roles played by other subjective dimensions of patient experience (the cognitive/thinking and the emotional/feeling components) in orienting PE towards the care process. The article suggests a possible framework to deeply understand the PE process useful to orient really attuned actions to support it. These results suggest the importance of developing patient engagement assessment tools that are more firmly grounded in the individual patient experience.

## Background

Patient engagement is recognized as a crucial component of high-quality healthcare services, particularly as far as chronic diseases are concerned [1,2]. In the majority of Western countries, the engagement of patients in the management of their health is well established, with governmental backing, and prioritizes the “patient’s active roles in their own healthcare” [3,4]. Zuckerman and colleagues [5] underlined the pivotal role of patient engagement in improving effectiveness and efficiency of care. According to their perspective, patient engagement is a pivotal element for turning individuals into co-producers of their health and enhancing their care experience, which results in improved health outcomes and lower healthcare costs. The risks of having disengaged patients, on the other hand, include the waste of healthcare resources and poor clinical outcomes [6]. There is a growing understanding of the critical role that engagement plays in improving health behaviors and clinical outcomes, which has prompted the healthcare industry to search for innovative ways to foster the role of the individual in the care process [5]. Patient engagement may lead to more responsive services and better care by incorporating the patient’s values and preferences into the care plans [7]. However, as Shortell [8] highlights, the healthcare system often fails to deliver effective interventions that are able to sustain patient engagement and bridge the gap between health and healthcare. Therefore, despite the growing attention on the role of patient engagement in the management of chronic diseases, up to now, a shared, evidence-based model has yet to be developed and, consequently, concrete guidelines for practice have not yet been determined [1]. The main issues with the integration of patient engagement are as follows:

–On the one hand, scientific contributions dealing with patient engagement consider this topic to be synonymous with other well established concepts, such as patient participation in healthcare plan and design [9], patient involvement in health/clinical interventions [10-12], patient adherence and compliance to treatment [13], patient activation and empowerment to enact healthy behaviors [14], and the therapeutic alliance in clinical encounters [15];

–On the other hand, these past studies mainly focus on the prospective outcomes of patient engagement in terms of clinical results [16,17] and organizational/economic advantages [18], while paying less attention to the development aspect of patient engagement.

–Finally, the study of the patients’ subjective experience of being engaged in the process of care (and what actions foster this experience) has been, so far, neglected. It is interesting to note, that the patient him/herself is the “great absent” in the discussion about patient engagement [19].

From our perspective, this lack of an evidence-based, theoretical and conceptual foundation, based on the patients’ direct experience, puts the healthcare industry at risk of losing the opportunity to nurture new innovations and to improve healthcare services and policies. We therefore advocate for empirical studies that are devoted to the identification of the elements that hinder (or foster) patient engagement.

### Disengagement consequences in type 2 diabetes

In this paper, we chose to focus on a prototypical clinical condition, in which the disengagement of patients in their own care is particularly challenging, namely, type 2 diabetes [20].

Diabetes affects 347 million people worldwide. Ninety percent of these have adult-onset, or type 2 diabetes. This number is likely to more than double by 2030, without effective intervention [21]. In order to keep the disease under control and to avoid diabetes sequelae, such as retinopathy and neuropathy [22], diabetics generally need to make numerous behavioral changes in areas such as diet, physical activity and adherence to treatment [23]. This means that these patients must be active and attentive in their daily care [24].

However, despite well-established clinical guidelines, the majority of diabetic patients struggle with managing their diet, physical activity and glucose self-monitoring [21]. Diabetes risk factors are modifiable with healthy behavior change but, sadly, rates of uncontrolled disease are high, both, from lack on adherence to initial oral drug treatment and in the long-term use of insulin [22]. The excessive numbers of patients with Type 2 Diabetes who are not achieving target levels of Glycated Hemoglobin (HbA1c) suggest that there is still a significant disparity between knowledge, understanding and effective health management [23]. Consequently, there is an unavoidable need to shift our focus and to look at new ways of managing diabetic patients, in order to better engage them in their care [24]. However, there is a lack of shared guidelines that could help the healthcare industry to reach this goal. With these assumptions in mind, this paper discusses the results of an in-depth qualitative study, designed according to a narrative inquiry approach [25,26], aimed at furthering:

–The subjective experience of uncontrolled type-2 diabetic patients in their care process, in order to explore the reasons for their disengagement, including the subjective dimensions of their experience and

–The elements (linked to health interventions, the healthcare system and the socio-cultural frame in which the patient is involved), which may hinder (or foster) the development of patient engagement in their care process.

## Methods

A qualitative health research design was used to identify, explore and explain complex attitudes and experiences of type-2 diabetic patients in managing their own care in daily life. A qualitative research design was chosen to conduct this study because it is particularly suited to grasp the complexities of psychosocial phenomena, such as patient disengagement in healthcare. The research design was developed according to the narrative inquiry approach [27]. Narrative reflection promotes introspection and self-reflection [28]. The primary goal of this approach is to reconstruct and collect insights on the sense-making process of getting ill, being ill, getting better (or worse) and coping (or failing to cope) with illness [29]. Among the different technical options offered by the narrative approach, we chose diaries and in-depth interviews [27] to collect data. Patient diaries and interviews were conceived as unstructured stimuli, able to elicit the free expression of an inner agenda of needs, expectations and priorities related to the illness experience. Diaries and interviews also allowed patients to reflect on significant aspects of their lives, as well as serving as a vehicle for construction, reconstruction and narration of their illness pathway and stories [30].

### Data collection

Diabetic patients registered in the study were required to keep a one-week diary related to their illness experience and disease management. Diaries were collected from April to May 2012. The diary was structured with two sections. The first was aimed at eliciting spontaneous narratives about the illness experience, focusing on the moment of the diagnosis disclosure and on the beginning of treatments. The second section was conceived as a daily-entry diary, where the patients were asked to synthesize the main events of each day (the more relevant items, in their perspective), to upload pictures expressing their feelings and to synthesize in one sentence of five words the “lesson learnt”. After the week of diary compilation, all patients participated in a face-to-face interview. The interviews lasted about one hour and were conducted according to a semi-structured guide, aimed at elucidating insights that had previously been collected through the diaries and at better understanding the meaning of the patients’ engagement in their care process. The interview began with the question ‘Can you tell me about your illness experience?’ Thereafter, the participants were encouraged to tell their illness stories. The interviewer used probing questions to encourage the participants to talk and to develop their stories. Questions about self-management were brought up in every interview (e.g., “How do you manage your illness? What do you do to manage your symptoms?”). Finally, questions about perceived positive and negative aspects to manage their disease and patients’ unmet needs were asked (e.g. can you tell me what are the main challenges in managing your illness? What could you need for better coping with your health condition?). An interview guide that was intentionally undetailed in order to facilitate patients’ expression (see Table 1) was used to conduct the interviews.

**Table 1:** Interview guide

**Area**	**Question**
**Living with diabetes**	*1. Let’s introduce your disease as if it was a human being, let’s describe how it appears, how it behaves and what it says….; let’s tell us the story of your illness: please feel free of deciding what to say and how to say it…but please imagine to tell a story to a child…elements can be real or fantasy based as far as they represent your experience*;
**The main turning points of the disease course**	*2. What are, if existed, the main events that features your illness journey?*
**Perceived positive and negative aspects to cope with diabetes**	*3. Overall, how well do you feel and think you are able to manage your diabetes?*
	*4. What were your difficulties with having diabetes over the past year?*
**Self-management practice**	*5. How do you manage your illness?*
	*6. What do you do to manage your symptoms?*
	*7. Describe your overall experience living with and managing diabetes”.*
**Patients’ unmet care needs**	*8. What kinds of support and resources would be most helpful to you in managing your diabetes?*
	*9. How would you improve the health services devoted to diabetes care?*

### Participant

Twenty-nine Type-2, uncontrolled (HbA1c > 8) diabetic Italian patients were involved in the study and were recruited through a snowball sampling strategy. The inclusion criteria were: patients had diagnosed with type-2 diabetes; were in treatment for at least 24 months of being enrolled in the study (thus being able to describe their experience in a vivid and detailed way); were at older than 18 years; were Italian-speaking; and were intellectually, emotionally, and physically capable of undergoing a clinical-psychological research interview; and had agreed to participate in the study (signed a declaration of consent). The intention was to include individuals with diverse types and experiences of treatment (oral treatment vs insulin treatment) and with a level of HbA1c > 8 in order to obtain insights upon the features of the participants’ disengagement experiences, as well as different illness trajectories. The sampling method utilized in qualitative research designs involves the intentional and purposive search for individuals who have information about the matter in question and are able to deeply articulate it. Data is produced with the purpose of reformulating, deflecting, complementing and/or helping to clarify initial assumptions, as is desirable in any scientific construction. The recruitment took place over a one-month period (March 2012) and was undertaken by approaching patients on the basis of a snowball sampling in collaboration with some general practitioners. We decided to focus on patients with uncontrolled diabetes, because the most critical cases show more dramatic extremes in furthering the analysis of the elements that may hinder or foster patient engagement. The sample was selected considering the type of therapy that the patient was undergoing: 16 patients were treated with two oral anti-diabetic drugs (OAD) and 13 patients had been treated with insulin for at least 2 years. Further sample characteristics are located in Table 2.

**Table 2:** Participant characteristics

**Characteristics**	**Number (N = 29)**	**%**	**Mean (DS) (range), year**
Age			51 (8.3) (41–71)
Sex			
Female	13	44.8	
Male	16	52.2	
Current treatment			
Oral medication	16	52.2	
Insulin	13	44.8	
Geographic origin			
Northern Italy	10	34.5	
Central Italy	9	31	
Southern Italy	10	34.5	
Living status			
Living alone	3	10	
Married empty nest	14	48	
Married full nest	12	42	
Educational status			
Middle high school	9	31	
Higher school	13	45	
University	7	24	
Work condition			
Retired	12	42	
Employed	10	34	
Never employed	7	24	

### Data analysis

In accordance with the qualitative narrative approach, the elicited narratives and interviews were subjected to an interpretive content analysis [27], by identifying associations between themes and carrying out an in-depth exploration of the emergent findings. All diaries and interviews were transcribed and analyzed using N-Vivo software for qualitative data analysis [31]. N-vivo is a “theory building software” [32]. This kind of software allows researchers to storage and manage data in a systematic way. In particular, it allows to make simpler coding, sorting, retrieving, comparing and integrating the data.

Two members of the research team double-analyzed a subset of the transcripts; disagreements and insights were discussed and alternative interpretations were incorporated in the analysis. The analysis team used an iterative process to discuss themes, clarify and expand upon interpretation of findings, and contextualize the coded responses. Through this process, the research team identified the main themes grounded in the data [27].

### Ethics statement

This study was conducted according to the guidelines laid down in the Declaration of Helsinki and quotes from the interview transcripts that might identify subjects were masked to protect confidentiality.

As per approved procedure, verbal or written consent was obtained from participants. Written consent was obtained in order to proceed with the diary and interview analysis. The patients in the study were made aware of the study goals and the privacy of all participants has been protected.

## Results

### Patient attitudes towards diabetes: reasons for being disengaged in care

See Table 3 for our findings from the analysis of the attitudes of uncontrolled Type-2 diabetic patients towards their disease and reasons for their lack of engagement in healthy behaviors.

**Table 3:** Impact of patients’ attitudes towards diabetes on the spheres of daily life, crucial in the management of the disease

		**Speheres of daily life**			
		**Diet**	**Physical activity**	**Therapy**	**Doctor-patient relationship**
Attitudes towards diabetes	Cognitive and informative barriers in diabetes management *(Cognitive dimension)*	The patients have difficulty understanding the rationale of the diet regimens prescribed by the doctor.	This is the area in which the patients seem to have less knowledge, or at least a less elaborate understanding of medical prescriptions, that often are perceived as abstract and outside of their daily context.	The patient reports an abstract knowledge of the therapeutic regime that he/she has to follow. Often he/she doesn’t understand the rationale behind the prescribed therapeutic scheme and he/she hasn’t interiorized the importance of adherence.	Information given from the doctor to the patient often appears partial. Educational and informative supports are often ineffective. As a consequence, the patient reports a fragmented knowledge about his/her status and the rationale behind the doctor’s requirements.
	The Behavioral Disorganization *(Behavioral dimension)*	Even in the case of a “cognitive adhesion” to diet prescription, the patients often report difficulty in translating treatment into the concrete frame of their daily life.	The majority of interviewed patients declare inconsistent physical activity. Physical activity does not often become part of patient routine and, rather, is rarely engaged in unless as a countermeasure for lack of adherence to diet.	The partial understanding of therapy rationale and values lead patient to unjustified “discounts” in drug assumption as well as to occasional “reparative” changes (i.e. increase) in the drug dosage.	The patient tends to “escape” the encounter with the doctor, by ignoring controls or by avoiding direct contact with the specialist.
	The Emotional Burden *(Emotional dimension)*	Food is strongly emotional and at the representational and symbolic levels, it not only allows the satisfaction of a primary need, but is also a source of gratification at the relational (conviviality) and individual levels (hedonism).	At the emotive level, this sphere is poorly invested; physical activity is insufficiently gratifying for the patient, and thus it is perceived as ancillary, less important than other medical prescription in the care process.	Therapy is treated with emotional ambivalence and conflict in patient experience. The reliance on drugs is s a constant reminder of the patient’s illness status, thus lack of adherence to treatment is often a sign of the patient’s reluctance to accept the awareness of his/her pathological status.	The doctor is ambivalently considered to be the most important point of reference for the patient, and at the same time as far away figure, poorly attuned to patient needs and priorities. Further the patient often - at the symbolic level – blames the doctor as the “executioner” who communicated the diagnosis, and thus dramatically changed the patient’s life.
				This is particularly evident in the case of insulin, lived as the “very end” of one own health status.	

#### Lack of knowledge

About the half of the sample (with a slight majority among patients following oral treatment) reported doubts and held superficial knowledge related to their health condition and their treatment.

This lack of knowledge appears attributable to the three “domains of experience”, which are crucial – in the subjective representation of the patient – for the daily management of their disease: diet, physical activity and pharmaceutical treatment.

Patients complained that doctors would often give “abstract” rules related to their treatment and lifestyle changes, which they had trouble integrating into their daily life.

“The doctor told me that I have to do some physical activity, but he didn’t advise on the kind of exercises I should do.” (male, 57 years old, oral therapy)

It follows that patients often elaborate “misconceptions” about their medical prescriptions and, often, are not aware of the inadequacy of their behaviors.

*“I normally engage in physical activity, since I walk to do my grocery shopping, for about 10 minutes a day”* (female, 65 years old, oral therapy)

*“I do drink wine every night, all of our ancestors drank wine without problems!”* (male,71 years old, insulin)

*“Sometimes I eat a bit more, although I know that is not right, but when I do, I’ll do some physical exercises to balance the extra that I ate.”* (male, 62 years old, oral therapy)

Even in the case of the most health literate patients, their knowledge appeared to be more abstract than concrete, and patients seem to have insufficiently understood the rationale behind medical requirements.

*“I try to be compliant with all my doctor said, but I confess that I didn’t really understand the sense of some prescription, for instance about the diet”* (female, 58 years old, insulin therapy)

#### Emotional burden

Moreover, patients generally describe diabetes as an intrusive and wearing presence, which is often thought of as a sly condition that makes the patient feel like a slave. Moreover, all patients reported feelings of anxiety and anger and described their disease as “dirty” and “binding”.

“[My diabetes] is like a tax collector, pushy, arrogant, always present…it has persecuted me for four years and still does.” (male, 41 years old, oral therapy)

“When I think about my diabetes, it makes me feel like a “slave”, slave of insulin pens, of finger pricks, of limitations “(female, 68 years old, insulin therapy)

The disease is described with a loss of freedom and as a condition from which patients occasionally try to escape, often resulting in failure to comply and engage in medical prescriptions.

“*My diabetes is like my conscience who punishes me when I make a mistake. It tells me: “I am like your shadow, don’t forget it.” (male, 61 years old, oral therapy)*

“On Saturday and Sunday, I engage in traditional dancing, because it allows me to forget my disease. During the weekend, I make some mistakes like drinking wine or eating sweets. But on Monday I start to follow the diet, again!” (male, 57 years old, insulin therapy)

It is interesting to note that patients, both in their diaries and in the interviews, describe themselves using words such as “*ill person*” and “*diabetic*”. This rhetorical tendency testifies to the high psychological impact of diabetes on individual identity and self- image, not only on the physical, but particularly on the psychological level. This health condition tends to make patients feel more like patients, rather than persons, due to its totalizing effect on the individuals’ life.

“[…]Also, when you are in good company, with family and friends and you are having a dinner you are forced to think of “IT”.” (female, 66 years old, oral therapy)

In other words, after the diagnosis, the self-perception of the patient seems to change, moving towards a configuration of their identity that deals mainly in terms of absence/negation, of “who they were” and “what they were able to do”, before the diagnosis. Diabetes involves a deep sense of fragility and vulnerability, which can be very hard to accept.

#### Behavioral disorganization

As mentioned above, even in the cases of “cognitive adhesion” to medical treatments, patients often report difficulties in translating these therapies into the structured frame of their daily life. This is often the case with dietary restriction, in regards to which patients would like to receive specific recipes and examples of how to concretely manage their therapy. Illustrations and enumerated rules would be of particular use in how to manage the evolution of daily routines. Until now, due to the scarce understanding of the reasons behind abstract medical prescriptions of therapy and lifestyle change, patients have displayed disorganized behaviors that have often failed to insure the efficacy of treatment.

“When you travel, it is very difficult to take the drug, it is also embarrassing.” (male, 68 years old, insulin)

“[…]Sometimes I forget to take my pills, so the day after I take a bit more of the drug in order to balance.” (male, 57 years old, oral therapy)

It follows that, in the majority of cases, (in particular among insulin treated patients), individuals appear to be behaviorally disorganized and unable to translate information received by their doctor in reproducible and effective behavioral practices to manage their health and treatment. In particular, they show an incomplete understanding of the reasons behind the need for following the therapy and complying with a new lifestyle regime.

Even the doctor-patient relationship sometimes appears challenging and patients tend to avoid contact with the diabetologist and to “forget” clinical check-up and doctors’ appointments.

“Honestly I hate the controls: my doctor is usually in a hurry, and I don’t feel like asking for more details. I feel stupid to ask.” (female, 60 years old, oral therapy)

**Figure 1:**
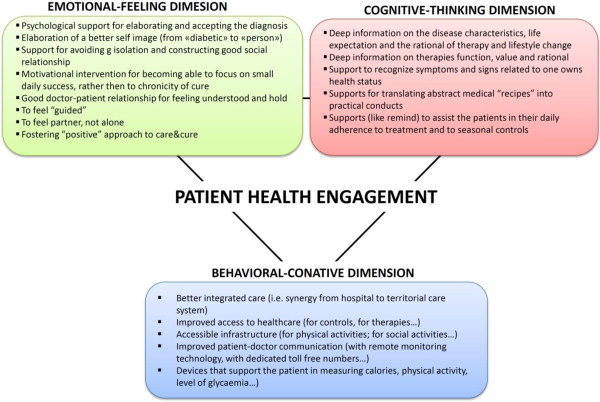
Factors fostering patient health engagement.

*“Sometimes I ignore the appointments and I don’t go.” (male, 47 years old, oral therapy)*Consequently, patients are not autonomous in the management of their care or their newly demanding life style, and therefore often fail to comply with them. Figure 1 shows the main actions that healthcare providers may deliver to support patients in overcoming the difficulties experienced in the informative, emotional and behavioral areas.

### The 4 experiential phases in the patient engagement continuum

Considering this framework, the issues/problems that a diabetic patient has to face can be connected to three main experiential dimensions:

•**Emotional dimension (“feel”)**: connected to the need to psychologically and emotionally manage (and accept) the onset of the disease and new life condition linked to it;

•**Cognitive dimension (“think”)**: connected to the need to know, understand and make sense of: the disease, its treatments, its possible developments, its monitoring;

•**Conative dimension (“act”):** connected to all the activities the patient acts out to face the disease, basing on its emotional and cognitive reaction

The synergy among these three dimensions structures four different patients’ experiential positions featured by specific unmet needs that patients experience in several domains of their daily life. These positions may be organized along a continuum from “blackout to eudaimonic project”.

#### The blackout phase

Patients in this position feel blocked and can’t accept the diagnosis and their new health condition: in this phase the patients deny the diagnosis, appear emotionally “frozen”, and totally unequipped in term of knowledge and information about their disease and the strategy to manage it (treatments, changes in life style…). These patients are neo-diagnosed and are cared by a general practitioners and not by a specialized diabetes center. These patients often feel alone in the face of the enormity of their disease, and they fail to completely accept their diagnosis, thus resulting in emotional barriers and other difficulties in truly engaging in their own health management.

“It is difficult to accept being ill, or at least it has been very hard in the beginning. You feel alone, no one understanding you. I would definitely have appreciated some counseling.” (man, 53 years old, oral therapy)

The main need for this patients is to answer to the question “Who am I?”; this because the disease onset asks patients to reframe their psychological image and to accept this new image of themselves. Supporting the patient in fostering a good self-image, one that is not reduced to the sole identity of being a patient, is needed. This approach would help patients to feel more socially included and to earlier come back to their daily life by figuring out how to live in a “new normality”.

“*The others just treat you as a patient, you are not a person anymore.” (female, 51 years old, oral therapy).*

#### The arousal phase

In this position patients had emotionally accepted the diabetes as a new self-dimension, but they aren’t able to adequately process (and learn) strategies for living with diabetes (changes in food, physical activity…) Resulting from this type of reaction, the subject lives is pathological experience as strongly isolating. Food often becomes an obsession as it is also a social sharing moment

“W*hen I go out with my friends for supper, I prefer to ignore my therapy. It is awful to stop and say: “Now I need to take my insulin”. I’d better stay at home by myself” (male, 67 years old, insulin)*

They are really terrified by diabetes and its possible consequences and they feel really alone and unable to identify possible reference network (e.g.: other patients).

A primary area of need concerns health literacy and the expectation of receiving more detailed information from the doctors and healthcare system, in order to fully understand their health conditions, the related risks and their life expectations. The patients, themselves, also advocate for more in relation to the prescribed therapy and the necessity there of.

“I think I need to understand better why I need to take this therapy and for how long…many questions pop in my mind and sometimes I search the Internet in order get answers.” (male, 55 years old, oral therapy)

“Of course I know that I have diabetes and I know that is a very terrible thing. I know that I have to follow many rules and to behave correctly, but sometimes I get doubts that I don’t know how to solve. I have periodical meetings with the doctor, but I need a more direct way of communication…such as a 24-hour number I can call, in case I need it” (female, 45 years old, oral therapy)

Furthermore, patients agree on the need for getting support aimed at helping them in translating their abstract knowledge into actual behavioral practices. In the patients’ experience, the doctor often gives “abstract” information related to their treatment and prescribed lifestyle, but there is no opportunity to educate patients in mastering necessary health skills Furthermore, patients advocated for concrete examples and testimonials from other patients, in order to figure out how to apply the guidelines to their daily life.

“The point is: how can you really comply with these rules in daily life…they are so abstract. I would need more concrete examples.” (male, 70 years old, insulin therapy)

“Once I attended an interactive session where the diabetologist, together with the dietician presented examples of new lifestyle rules, It was nice: with videos and patient interviews. I think that something like that could be very useful and should be repeated!” (female, 47 years old, oral therapy)

More over these patients requested, opportunities for networking and meetings with patients that share the same clinical experience. This would allow for the sharing of best practices and “situated solutions” to solve the difficulties faced in the daily management of diet, physical activity and drug adherence. This may also allow patients to manage their treatment and prevent avoidable complications, while maintaining or improving their quality of life.

“It would be nice to have dedicated spaces in which to meet with other patients to do activities together. Like cooking classes or physical activity programs. This would be very useful to feel connected and less lonely, but also to receive concrete suggestions to manage your disease.” (female, 64 years old, oral therapy)

“I don’t think that any dedicated structures for diabetic patients exist; this is the problem!” (male, 65 years old, insulin therapy)

#### The adhesion phase

These patients adequately answer to the physician prescriptions but they can’t emotionally accept their lifestyle changes. They need to answer the question “why do I do this?”. Food, physical activity and self-monitoring prescriptions are external norms the meaning is unclear for patients.

They need an adequate motivation at the emotional level in order to make sense to the changes they are doing in their daily life.

These patients need to be empowered and helped to feel “successful” again, by focusing on small goals, which will encourage them on to greater success.

“I feel tired and without motivation. When I am in control, I achieve better results. In that case, I feel extremely happy and I feel that I can succeed in managing the disease. I think motivation is the key. It is hard, but I have to find motivation” (female, 49 years old, oral therapy)

The doctor-patient relationship appears to be a key in offering the patients the emotional support they need. However the patients in our study reported unsatisfactory relationships with their doctors, who were often perceived as too harried, and described the consultations as lost opportunities to receive support. On the contrary, they would like to feel that their doctors come alongside them and engage in a partnership relationship, thus establishing an effective collaboration. According to patient narratives, the doctor-patient relationship is fundamental in order to develop a positive approach to treatment and to really understand what they need to know how to safeguard their health.

“My doctor is good, but he doesn’t actually have time for engaging in a real conversation. I would appreciate having the opportunity for a more relaxed encounter with him.” (male, 65 years old, insulin therapy)

“I don’t feel very supported by my doctor. I do not feel like I could express my feelings, my difficulties, I do not think he would understand. However, I think that I would love to have someone who could coach me and motivate me in the daily life of my therapy.” (female, 57 years old, oral therapy)

#### The eudaimonic project phase

These patients have been able to set up an adequate cognitive and emotional answer to diabetes. They accepted their disease and understood it doesn’t impede to continue their relational and social life and also the importance and the sense of their cure and their lifestyle changes.

Needs is focused on specific aspects of the care (really connected to their practical experience).

Patients expressed unmet needs and expectations related to a venue to access the healthcare system from home, for instance by using new technologies and remote communication. In their view, this might improve the possibility of receiving instant and on-demand feedbacks when the patient is enacting a healthy behavior but he/she feels insecure.

“It would be useful to have a mobile tool, or a website that you can use whenever you need, to communicate with your doctor or with the hospital. Actually, up to now, if you have a doubt about your conduct, you don’t know to whom to ask.” (male, 63 years old, oral therapy)

“I would like to have a remote control, a kind of telemedicine tool to feel controlled and supported every day. I think this would help in motivating me.” (female, 55 years old, oral therapy).

## Discussion and conclusion

Although the present study was based on a relatively small sample size, the qualitative approach that we used provided a broad spectrum of insights to further engagement of Type-2 diabetic patients and enable the identification of factors that might contribute to fostering (or hindering) this experience.

In particular, our results suggest that patient health engagement results from the conjoint cognitive, emotional and conative response of individuals towards their health condition. The inability to achieve synergy among these subjective dimensions inhibits patients’ ability to engage in their own care and, consequently, to obtain the greatest benefit from the healthcare systems in terms of health, wellbeing and a sustainably healthy life style.

Furthermore our results suggest a process like nature of the engagement experience, that cannot be reduced to a simpler polar activation (in the logic “on”/”off”).

Particularly, the study underlined that engagement evolve throughout four progressive patient’s position resulting from the synergic interlacement of the three constitutive domains of patients experiences (“*think*”, “*feel*”, “*act*”).

These experiential dimensions play complementary driving roles, as key factors for promoting patients’ advancement in their health engagement process. The unachieved synergy among these different subjective dimensions inhibits patients from effectively engaging in their health management. Specifically, the process of patient engagement develops in four sequential phases:

1) The pre-phase of disengagement, at the onset of a new health status, where patients feel blocked and at the mercy of the healthcare system: in this phase the patients deny the diagnosis, appear emotionally “frozen”, incapable of understanding their health condition, and are therefore not equipped to handle health management. In this phase it is crucial to help patients to became aware of the problem and to provide an initial knowledge toolbox to kick off their mobilization to health management. 2) A more proper phase of engagement follows, where the patients begin to perceive themselves as “ill” but still delegate the majority of responsibilities for their health management to the healthcare system. At the emotional level, the patients are aware of their condition and too hurried about their status, but they still do not have enough cognitive/informative elements to attribute sense to their health condition. This patients’ status provoke a behavioral disorganization in their health management. To sustain the evolution to the following phase, it is crucial to offer tailored psychological support to patients and their caregivers to assist them in elaborating the diagnosis and in enacting finalized health behaviors. 3) A phase of formal “adhesion” to the medical prescription follows. In this phase, the patients have finally accepted the diagnosis and obtained sufficient abstract knowledge about the disease and its treatment. However, the patients appear unable to fully comprehend their prescriptions and to manage them in autonomy. Patients thus become formally adherent to treatment, on the basis of a rigid “script”. To move forward in the process and to become more autonomous in the management of their health, the patients need to really understand the rationale behind their medical prescriptions. In this way patient can successfully participate in the health decision-making process. 4) Finally, a phase of full engagement follows in which patients become co-producers of their health. In this phase the patients succeed in considering themselves as “person” where the illness condition is only one of the multiple life domains in which they are involved (eudaimonic project phase). To sustain this adaptive psychological status, individuals need for continuous counseling and targeted information to empower their ability to co-produce their health.

These results need further validation, but they are a first step toward the empirical foundation of a theoretical framework of patient health engagement. Firstly, the patients’ descriptions of the three synergic dimensions of patient engagement, although not new in the scope of health psychology, offer a valid reason to debate the adequacy of actual definitions of patient engagement, that so far seem to focus only on single aspects of these subjective dimensions, without acknowledging their interdependency [3,33,34]. Until now, wider and holistic explanations of patient engagement have been absent and the most cited definitions in the academic literature still appear poorly grounded in the study of patient experience. In particular, Carman and colleagues [35] define patient engagement as “a set of behaviors by patients, family members, and health professionals and a set of organizational policies and procedures that foster both the inclusion of patients and family members as active members of the health care team and collaborative partnerships with providers and provider organizations with the desired goals of patient and family engagement include improving the quality and safety of health care.” This definition of patient engagement has the indubitable strength of considering engagement as a systemic concept, which is the outcome of actions carried out at different levels of complexity (i.e. individual, relational, communitarian, organizational and health policy). However, this definition is insufficient, in as much as it reduces the engagement process to merely the behavioral/conative dimensions of patient experience. On the contrary, according to our study, the behavioral dimension of engagement is only one of those implicit in the process and often depends on a patient’s position in the other two experiential levels (emotional and cognitive).

Additionally, other scholars define patient engagement in terms of level of “activation”, by defining an engaged patient as “an active agent in the management of his/her own health, including developmental stages “of 1) believing the patient role is important, 2) having the confidence and knowledge necessary to take action, 3) actually taking action to maintain and improve one’s health and 4) staying the course even under stress” [33]. This definition, which has played a crucial role in the discussion over patient engagement, and that appears to be the most complete and systematic descriptor, from our perspective, fails to recognize the multiplicity of the subjective dimensions acting behind the behaviors of the patient. Concepts such as “beliefs” and “confidence” related to patient experience concern much more than the emotional and cognitive aspects of the patient experience, thus confirming our findings.

Similarly, Gruman’s patient engagement behavioral framework [34] has the value of systematizing the different components of the engagement experience. In our experience, however, it fails in the identification of the psychological dimensions implied in the process: here again the behavioral component of patient engagement appears to be an outcome of the synergy among other subjective dimensions, rather than one of the psychological levels of the patient’s ability to function in the health engagement process.

In brief, our findings appear to confirm some themes detected by other scholars in their definition of patient engagement, particularly in relation to its conative-behavioral components. But they underline the importance of also considering other subjective dimensions of patient experience that seem to have a synergic (probably antecedent) role in determining patient activation and adherence to the health process and prescriptions. In particular, actual definitions of patient engagement do not fully consider the emotional (feeling) and cognitive (thinking) components of this experience, which, in our findings, appear to be crucial in understanding patient availability and capacity to engage in self-care. Furthermore present definitions of engagement fail in clarifying its progressive development and tend to reduce this experience to a “status” that can or cannot be achieved by the patient.

On the contrary we claim that a process-like conceptualization of patients’ engagement potentially may sustain the real innovation of healthcare paradigms in research and intervention, by providing a wider and more systemic vision of patients' experiences and preferences.

In this regard, the present emphasis on the “conative” component of patient engagement appears as a reparatory answer to the still “passivizing” approach of medical practice, within a health system that has not yet succeeded in the fulfillment of a truly patient-centered approach to the care [36]. Often the patient is still considered to be a “disease carrier” and the focus still highlights the treatment of disease, rather than the person as a whole. The adoption of a broader and more systemic conceptualization of patient engagement would lead to a more genuine consideration of patients as persons, who have histories, desires, needs, preferences and projects for their present and future lives: projects that – at least at the emotive level – should not become inconsequential because of diabetes, but – at most–be reconfigured and reoriented, in the eudemonic development of a new self-representation [37]. To have a sense of control over one’s own disease and cure process - not only at the behavioral level, but primarily at the cognitive and emotional levels – appears to be crucial for guaranteeing a true engagement of people towards their health and care [9]. In other words, this may call for a rewording of the term “patient engagement” to “personal health engagement”, in order to underline the importance to help patients become aware, accept and incorporate their disease (and its treatment) in a new, achievable and positive planning of one’s own health and wellbeing. Moreover, from our perspective, there is a growing need for research approaches that are able to give voice to the “intimate view of problems and needs” for each patient [38]. This would really promote care practices that are fine-tuned with the subjective experience of patient engagement and priorities In this arena, qualitative research can contribute substantially to the revision of healthcare practices in the aim of fostering better patient activation in their own health management. As our results suggest, focusing on the subjective experiences of patient illness, and of their own individual ways of engaging in health management, is becoming an indispensable component of healthcare research, and it may illuminate which models are most effective, thus fostering innovative interventions that can make the healthcare system more responsive to patient needs. Finally, our results also emphasize the urgency of developing assessment tools that are really attuned to the subjective dimensions of patient engagement (and not solely to the conative one: see, for instance, the Patient Activation Measure [33], in order to support a better customization of health interventions. These tools can help healthcare innovation – even health technology advancement [9,39-41]-based on an ecological understanding of patient preferences and priorities in the frame of a broader vision of health and wellbeing.

Engaging patients in their care remains a crucial issue in the treatment and management of type-2 diabetes, particularly for high-risk patients. In as much as diabetes management requires long-term adherence to complex regimens, the attitudes and the subjective experience of patients are of primary importance and must be taken into consideration. Further research should aim to gain a better understanding of the role of the health practitioner in facilitating patient engagement for effective disease management. We thus advocate for future research projects able to guarantee a deep understanding of the subjective patient engagement experience in order to sustain the shift from a “patient centered” to an actual “people oriented” approach to medicine. Therefore, we suggest to move from the consideration of individuals as merely “patients” to “persons” able to plan for their present and future life trajectories on the bases of their subjective experience of health engagement. “Persons” who want to “speak laud their voices” for orienting healthcare system approaches and priorities. “Persons” who need to be heard, understood, and considered for the innovation of healthcare systems as participants in their wellbeing achievement and eudaimonic expression of self-potentialities.

## Competing interest

The authors declare that they have no competing interests.

## Authors’ contributions

GG, SB and CL carried out the analysis and interpretation of data; CB participated in the design, revising it critically for important intellectual content and participated in the interpretation of data and final approval of the version to be published. All authors read and approved the final manuscript.

## Pre-publication history

The pre-publication history for this paper can be accessed here:

http://www.biomedcentral.com/1471-2458/14/648/prepub
